# Different Strains of Microalgae in a Microbial Consortium Remove Antibiotics from Urban Wastewater in Batch and Continuous Treatment Systems

**DOI:** 10.1021/acsomega.6c02775

**Published:** 2026-04-30

**Authors:** Sarah Regina Vargas, Julia de Lima Santos, Pedro Henrique Ferraz Ribeiro, Iliseu Monteiro Alcântara, Rodrigo Braz Carneiro, Marcelo Zaiat

**Affiliations:** † Laboratory of Microbiological Processes, Department of Biomedical Sciences and Health, 248092 Minas Gerais State University (UEMG), 164, Sabará St., Centro, Passos, Minas Gerais 37900-004, Brazil; ‡ Laboratory of Biological Processes, São Carlos School of Engineering, University of São Paulo, 1100, João Dagnone Ave., Santa Angelina, São Carlos, São Paulo 13563-120, Brazil; § Laboratory of Chromatography, São Carlos Institute of Chemistry, University of São Paulo (USP), 400, Trabalhador São-Carlense Ave., São Carlos, São Paulo 13566-590, Brazil

## Abstract

Antibiotics are micropollutants present in aquatic ecosystems, thus causing serious concerns. The presence of mixotrophic microalgae in wastewater biological treatment systems is important for the removal of those micropollutants. This study evaluated the removal efficiency of five different antimicrobials, sulfamethoxazole, trimethoprim, ofloxacin, ciprofloxacin, and enrofloxacin, in batch and continuous photobioreactors by four different Chlorophyceae strains in consortium with autochthonous bacteria. In a batch system, all strains efficiently removed the 5 antibiotics—mainly sulfamethoxazole, enrofloxacin, and ciprofloxacin by *Desmodesmus* sp. (86 ± 2% average removal) and *Chlamydomonas* sp. (85 ± 1% average removal). In the continuous system, *Chlorella* sp. also removed the 5 antibiotics, but with better results for sulfamethoxazole and trimethoprim (54 ± 2% and 57 ± 4% average removals, respectively). In the batch system, the highest removal efficiencies were achieved by *Chlamydomonas reinhardtii* for organic carbon (95 ± 1%) and nitrogen (89 ± 1%) and by *Desmodesmus* sp. for inorganic phosphorus (91 ± 6%), whereas in the continuous system, 97% organic carbon were removed by *Chlorella* sp., and better removal performance for four of the antibiotics compared to the batch system. Therefore, microalgae-bacteria consortia are effective for wastewater biological treatment with antimicrobials, a sustainable treatment alternative. Furthermore, this process contributes to the valorization of residues by simultaneously allowing the production of byproducts, such as organic acids and biohydrogen.

## Graphical abstract

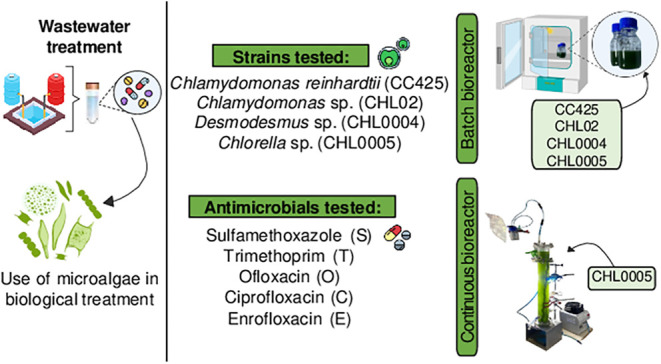

## 1 Introduction

Pharmaceutical compounds are organic micropollutants found in wastewater systems from various sources, such as domestic, hospital, agricultural, and pharmaceutical effluents.^1^ Those medicine products are complex organic aromatic molecules with different functional groups and several physicochemical and biological properties, which are also essential for human and animal health. Due to the operation time required by pharmaceuticals for acting in the organisms’ treatments, they must display persistent characteristics for maintaining their chemical properties.

Several antimicrobial subdivisions are produced in society (e.g., penicillin, tetracyclines, quinolones, sulfonamides, lincosamides, fluoroquinolones, glycopeptides, and acrolides, among others), differing in terms of target microorganism.^2^ Regarding sulfonamides and fluoroquinolones, the former are constantly detected in effluents due to their strong persistence and low biodegradability^3^ and are partially metabolized (50% to 80% are excreted through feces and urine and enter from domestic sewage).^4^ In other words, the organisms can eliminate most drugs and form free radicals, causing oxidative damage to cells.^5^

Therefore, the presence of antibiotics in aquatic ecosystems has been the cause of concern, since they are not completely degraded in the organisms and most of their active elements are eliminated in the excrements, contributing to the input of drugs into sewage systems.^4^ Furthermore, medicines have low biodegradability and a high bioaccumulation capability in individual bodies, leading to cellular mutations, selection of resistant pathogenic microorganisms, and an ecological imbalance over time.^6^

Besides the difficult natural degradation of those micropollutants in the environment, conventional physical-chemical treatments either cannot remove most of them due to a lack of specific methods for each type of pollutant in Wastewater Treatment Plant (WWTP), or are expensive, limiting their application.^1,6,7^ Toward alternative solutions to such an environmental health situation, studies have aimed at reducing the impacts of antimicrobial contamination on water bodies through, for example, biological treatments, which commonly use microorganisms such as bacteria, fungi, and microalgae for wastewater treatment. They are a promising alternative in function of their easy upkeep and low operating costs compared to other technologies.^8–11^

One of the possible biological treatments is the use of mixotrophic chlorophytes. Mixotrophic microalgae can consume organic and inorganic compounds from wastewater and remove micropollutants and have different metabolic pathways for providing value-added products.^12,13^ Another benefit is the existence of species of high growth rates, leading to productivity and efficiency in the treatment of several types of sewage effluents.^10,14^ Nevertheless, most investigations on the use of microalgae as a biological wastewater treatment derive from studies on the removal of organic matter and macronutrients such as phosphorus and nitrogen. Experiments with those microorganisms have been recently conducted toward biological treatments of hospital wastewater or pharmaceutical components (e.g., antibiotics, anti-inflammatories, and hormones) through microalgae (mainly chlorophytes such as *Chlorella*, *Chlamydomonas*, and *Scenedesmus*) in both batch and continuous systems, aerobic and anaerobic.^11,14–19^

Recent studies have demonstrated the removal of antibiotics by microalgae in both batch and continuous systems. In experiments using *Auxenochlorella protothecoides*, *Tetradesmus obliquus*, and *Chlamydomonas acidophila* for the removal of ciprofloxacin, clarithromycin, erythromycin, metronidazole, ofloxacin, sulfamethoxazole, and trimethoprim in batch assays at concentrations of 10, 50, and 100 μg L^–1^, removal efficiencies of up to 70% were observed for ciprofloxacin, clarithromycin, metronidazole, and ofloxacin, mainly by *A. protothecoides* and *C. acidophila*.^20^ Studies with other chlorophytes, such as *Monoraphidium contortum*, using a bench-scale tubular photobioreactor with 500 μg L^–1^ of sulfamethoxazole and trimethoprim, showed that *M. contortum* was able to remove 42.3% and 28.6% of these antibiotics, respectively.^21^

The microalgae possess different mechanisms for metabolizing these compounds, which vary among the different species of microalgae.^7,16^ The main removal mechanisms include biosorption, bioaccumulation, and both intracellular and extracellular biodegradations. Additionally, photodegradation, volatilization, and hydrolysis may also occur under specific conditions, although they contribute to a lesser extent to the overall removal process.^7,13^

Research has investigated the mechanisms of antibiotic removal by *Scenedesmus almeriensis*, including tetracycline, ciprofloxacin, sulfadiazine, and sulfamethoxazole. The results demonstrated that biosorption was the main mechanism for all four antibiotics, followed by biodegradation in the case of tetracycline and sulfadiazine. In addition, sulfamethoxazole showed no removal through hydrolysis or photolysis.^22^ Another study also reported that ciprofloxacin removal occurred mainly through biosorption by three different chlorophyte strains.^13^ Oliveira et al.^23^ demonstrated that the removal of sulfamethazine via hydrolysis, photolysis, volatilization, and adsorption onto the bioreactor walls was considered negligible.

However, one way to improve wastewater treatment efficiency is through the cultivation of microalgae in a consortium with autochthonous bacteria, since such consortium can occur naturally and intensify nutrient exchanges, provide resistance to environmental factors, and contribute to chlorophytes’ growth.^24–26^

Biological treatment through mixotrophic microalgae cultivated with autochthonous bacteria has been highlighted due to its environmental and economic viability. Studies indicate that the removal efficiency of antibiotics is higher in microalgae–bacteria consortia.^22,27^ The interactions between microalgae and bacteria, as well as the exchange of metabolites between these microorganisms, can lead to enhanced antibiotic degradation and nutrient removal in wastewater treatment through cometabolism, in which the simultaneous and interdependent degradation and transformation of different compounds occur.^27,28^

In this context, this study aimed to preliminarily evaluate the removal efficiency of five antimicrobials, sulfamethoxazole, trimethoprim, ofloxacin, ciprofloxacin, and enrofloxacin, which are commonly detected in wastewater,^29^ from synthetic wastewater by four different mixotrophic chlorophytes, two strains of *Chlamydomonas* sp., *Chlorella* sp., and *Desmodesmus* sp., in batch system photobioreactors, and *Chlorella* sp. in a continuous system. Three of these strains are wild, have never been previously studied for this purpose, and were isolated from wastewater in Brazil. The removal efficiencies of macronutrients, as well as the generation of byproducts such as organic acids and biogas, were also evaluated. It is hypothesized that these previously unstudied wild strains may represent promising and effective alternatives for the biological treatment of wastewater contaminated with widely used antibiotics and that the results will contribute to further investigations in the future.

## 2 Materials and Methods

### 2.1 Strains, Maintenance Conditions, and Growth Observation

Four chlorophytes’ strains, namely, *Chlamydomonas reinhardtii* (CC425 - cw15 arg2 sr-u-2–60 mt+), *Chlamydomonas* sp. (CHL02), *Chlorella* sp. (CHL0005), and *Desmodesmus* sp. (CHL0004) were used. CC425 was obtained from a biological bank of *Chlamydomonas Resource Center*, and the other strains were obtained from the microalgae bank of the Laboratory of Biotoxicology of Continental Waters and Effluents – EESC/USP (São Carlos). All strains were nonaxenic and their cultivation in synthetic wastewater that simulates sewage proposed in this study^30,31^ (adapted by Carneiro—Details in Supporting Information - SI A) supplied the growth of autochthones bacteria.

The strains were kept in glass test tubes with semiscrewed caps inside incubators under controlled temperature of 24 °C, 12 light:12 dark photoperiod, and light intensity of 60 μmol m^–2^ s^–1^, provided by LED lamps, pH 7.2, in the control growth medium of Tris Acetate Phosphate (TAP),^32^ and with arginine supplementation (100 mg L^–1^) for CC425. Culture replicates of the strains were performed monthly and aseptically in a 1:9 ratio in a previously autoclaved culture medium at 121 °C for 20 min.

The antimicrobials selected for this study were sulfamethoxazole, trimethoprim, ofloxacin, ciprofloxacin, and enrofloxacin, which are widely used and commonly detected in urban wastewater and effluents worldwide.^29^ Some of these compounds are also frequently included in international priority pollutant lists because of their persistence, biological activity, and high occurrence in freshwater, sediments, and even drinking water.^33^

An ecotoxicology experiment analyzed whether the synthetic wastewater and the five aforementioned antibiotics influenced the strains′ growth. Four different concentrations (100, 10,000, 50,000, and 100,000 μg L^–1^) of each antibiotic were solubilized in methanol and individually tested in 10 mL glass test tubes with semiscrew caps containing 5 mL of culture, triplicated, in TAP medium (control with methanol) and synthetic wastewater.^30,31^ The tubes were kept in the incubator at 30 °C, 12 light:12 dark photoperiod, 150 μmol m^–2^ s^–1^ light intensity (20 W LED lamps), and pH 8.0. The strains’ growth was monitored for 10 days by absorbance (680 nm)^34^ and no inhibition was observed under the tested conditions with 100 μg L^–1^ in synthetic wastewater (previous results in Supporting Information, SI B).

Additionally, the EC_50_ (effective concentration for 50%) was calculated to determine the minimum concentrations of the antibiotics for the four strains (SI B – Figure S5) according to Brain-Cousens (BC.4) and AIC (Akaike Information Criterion).^35,36^ The test was performed in triplicate for all antibiotics and concentrations mentioned above. The results obtained in the ecotoxicological assay contributed to the selection of the concentration used in the batch system antimicrobial removal test, estimating the maximum concentration at which these strains could be applied in a biological treatment for the removal of these compounds.

The growth of the strains throughout the antimicrobial removal assays in batch and continuous systems was evaluated with the use of indirect growth estimates based on turbidity at 680 nm,^34^ and the dry biomass was determined from total suspended solids according to APHA.^37^

### 2.2 Experimental Design of Assays of Antibiotics and Macronutrients Removal

In the first phase, biomass from all microalgae strains was prepared for the tests of antibiotics removal. The cultures’ volumes were higher in Duran flasks (2 L) for the batch system test and in Erlenmeyer (4 L) for the continuous system test under aerobic conditions inside the incubators under the same physical chemistry conditions as the ecotoxicology test (pH 8.0, 30 °C, 12 light:12 dark photoperiod, and 150 μmol m^–2^ s^–1^ light intensity). However, TAP medium was used for the obtaining of a larger amount of microalgae biomass.

In the second phase, tests of antimicrobial removal were conducted in a batch system with all strains in triplicate and in a continuous reactor (*Chlorella* sp.), with synthetic wastewater simulating lab-made wastewater with addition of antimicrobials.

Despite other studies reporting that the removal of certain antibiotics through hydrolysis, photolysis, and volatilization is negligible,^22,23^ an abiotic control was performed to evaluate the removal of the antibiotics sulfamethoxazole, trimethoprim, ofloxacin, ciprofloxacin, and enrofloxacin. This assessment was based on the analysis of initial and final concentrations after 10 days, according to the test conducted in batch systems, both in the presence of light and under dark conditions.

According to the results and statistical analyses, no significant difference was observed between the initial and final concentrations of all antibiotics, under both light and dark conditions, indicating that there was no removal of the antibiotics by hydrolysis or photolysis and that adsorption and volatilization were negligible (results presented in the Supporting Information, SI C).

#### 2.2.1 Removal Test in Batch Systems

Antibiotic and macronutrient removal tests in batch system photobioreactors were conducted in 250 mL Duran flasks with a total volume of 150 mL of culture, in triplicate, and with a semiscrewed cap in synthetic wastewater. The flasks were kept in incubators under the same conditions of the ecotoxicology tests (30 °C, 12 light:12 dark photoperiod, 150 μmol m^–2^ s^–1^ light intensity, and pH 8.0). Regarding the assembly of each photobioreactor, 150 mL of each strain’s biomass were measured under aseptic conditions in the exponential growth phase. The biomass had been prepared in the first phase and centrifuged for 8 min at 2.000 rpm in sterilized falcon bottles. It was then resuspended in Duran flasks with 150 mL of wastewater with the five antimicrobials previously tested, according to the results obtained in the ecotoxicological assay (100 μg L^–1^ solubilized in methanol). The test lasted 10 days and initial and final concentrations of micropollutants and macropollutants from the batch system were evaluated.

#### 2.2.2 Removal Test in the Continuous System

A photobioreactor was set in a continuous fixed bed system with ascending flow and polyurethane foam on rods as a support material for the adhesion of microorganisms, as proposed by Carneiro et al.^31^ A bag attached to the reactor caught the biogas produced during the reactor’s operation, which lasted 28 days. The total volume was 1.7 L and hydraulic retention time (HRT) was set to 48 h (0.59 mL min^–1^ theoretical flow rate) according to the generation time of *Chlorella* sp. The reactor operated in a locked room under environmental temperature (27 ± 2 °C), 12 light:12 dark photoperiod, 150 μmol m^–2^ s^–1^, and pH 8.0.

*Chlorella* sp. strain culture was inoculated into the reactor, as it exhibited a better growth rate during the ecotoxicological test in synthetic wastewater and higher biomass gain. Its volume was first increased, as described in Section 2.2, and transferred to the reactor in the middle of the exponential growth phase. After inoculation of the chlorophyte culture in the bioreactor, the inoculum was recirculated for 2 days for acclimatization. In the next step, this continuous system was opened and fed with a pump in a continuous flow by synthetic wastewater with the mix of the five antibiotics at 10 μg L^–1^ concentration, which is close to the one found in and commonly detected in urban wastewater and effluents,^1,29,33^ for 28 days. Twice a week, the reactor was fed and affluent and effluent were sampled. The flow rate was controlled daily to maintain the HRT adopted.

### 2.3 Assessment of Antimicrobials and Nutrients Removal and Generation of Byproducts

The efficiency of antimicrobials and macronutrient removal by biodegradation or absorption was analyzed through percentage of difference in initial and final concentrations of those compounds in both affluent and effluent collected from the continuous reactor twice weekly and difference in initial and final concentrations between days zero and day 10 of the photobioreactors in a batch system. The calculation of specific removal was based on percentage of difference between initial and final concentrations of those compounds and percentages per biomass (% removal by mg TSS^–1^). The samples were filtered through a 0.2 μm membrane for analyses.

The antimicrobials were quantified by an offline SPE system and liquid chromatography (Acquity UPLC) interfaced with mass spectrometry (XEVO TQ MS, Waters Corporation) (HPLC-MS/MS) according to Carneiro et al. and Lima Gomes et al.^38,39^ The aqueous mobile phase (A) consisted of ultrapure water containing formic acid 0.1% (v/v), and a solution of acetonitrile containing formic acid 0.1% (v/v) was applied as an organic mobile phase (B), at 0.35 mL min^–1^ flow rate. The other parameters used in HPLC-MS/MS can be consulted in Supporting Information (SI D).

Macronutrient analysis, total nitrogen, inorganic phosphorus (orthophosphate), and organic carbon (chemical oxygen demand, COD) were performed according to APHA,^37^ and the analysis of organic carbon by carbohydrate was conducted by the spectrophotometric colorimetric method.^40,41^ The average initial concentrations of nitrogen, orthophosphate, COD, and carbohydrates were 103.04 mg L^–1^, 11.28 mg L^–1^, 1051.75 mg L^–1^, and 320.26 mg L^–1^, respectively.

For the analysis of the generated byproducts, such as volatile organic acids, samples were collected twice a week from the continuous reactor and from the batch photobioreactors at the end of the test (10th day). The generation of volatile organic acids was detected by liquid chromatography in a SHIMADZU system with a SPDM10Avp UV detector with a 205 nm diode array and an Aminex HPX-87H ionic exchange column.^42^ Another analyzed byproduct was the biogas generated in the continuous reactor. The gas sample contained in the bag at the end of the reactor operation was analyzed by gas chromatography.^43^

### 2.4 Data Analysis

For the EC_50_ analyses, the R Project for Statistical Computing software was used. The *Kolmogorov–Smirnov* normality test analyzed the normality of data. Throughout the confirmation of the normal distribution of the results, T-*student* and analysis of variance (ANOVA) followed by the Tukey’s *post hoc* test verified possible statistical differences in the removals of antimicrobials and macronutrients. *GraphPad Prism* 8 statistical software was used and *p* ≤ 0.05 indicated a statistically significant difference.

## 3 Results and Discussion

The preliminary results of the ecotoxicological test showed the strains investigated exerted no influence on the growth of the strains in the synthetic wastewater under the proposed physical-chemical conditions and in the 100 μg L^–1^ concentration of the 5 antibiotics (results in Supporting Information – SI B), promoting antimicrobial and nutrient removal tests in continuous and batch systems.

The results of the EC_50_ analysis (Figure S5) indicated that *Chlorella sp.* (CHL0005) was the most resistant strain to antibiotics, particularly to sulfamethoxazole, ofloxacin, and enrofloxacin (EC_50_ ≥ 96.78 mg L^–1^), while it was more sensitive to ciprofloxacin (EC_50_ = 3.59 mg L^–1^). CHL02 exhibited greater sensitivity to sulfamethoxazole (EC_50_ = 1.11 mg L^–1^), whereas CC425 and CHL0004 were more resistant to this antibiotic (EC_50_ = 100 mg L^–1^). CC425 and CHL0004 showed higher sensitivity to enrofloxacin (EC_50_ = 0.98 mg L^–1^) and ciprofloxacin (EC_50_ = 1.02 mg L^–1^), respectively.

Positive results were obtained with all chlorophyte strains under study, i.e., *Chlamydomonas* sp., *Chlorella* sp., and *Desmodesmus* sp., regarding efficiency in nutrients and antimicrobials removal in both batch and continuous systems by *Chlorella* sp. Although the results of this investigation are preliminary, since these strains have not been previously studied, the data may be applied in future research, particularly in studies focusing on the mechanisms of antibiotic removal by these strains and on the cometabolism within the microalgae–bacteria consortium.

### 3.1 Antibiotics Removal

#### 3.1.1 Tests in the Batch System

The initial average biomasses of *C. reinhardtii* (CC425), *Chlamydomonas* sp. (CHL02), *Chlorella* sp. (CHL0004), and *Desmodesmus* sp. (CHL0005) in the photobioreactors were 139.55 ± 24.72 mg L^–1^, 171.61 ± 29.06 mg L^–1^, 543.33 ± 23.57 mg L^–1^, and 300.00 ± 14.14 mg L^–1^, respectively. According to the results, all strains showed a significant removal percentage for the 5 antimicrobials tested in batches. On average, antibiotics sulfamethoxazole, enrofloxacin, and ciprofloxacin showed the highest removal efficiencies by the microbial consortia, whereas ofloxacin and trimethoprim achieved the lowest (Figure 1).

**Figure 1:**
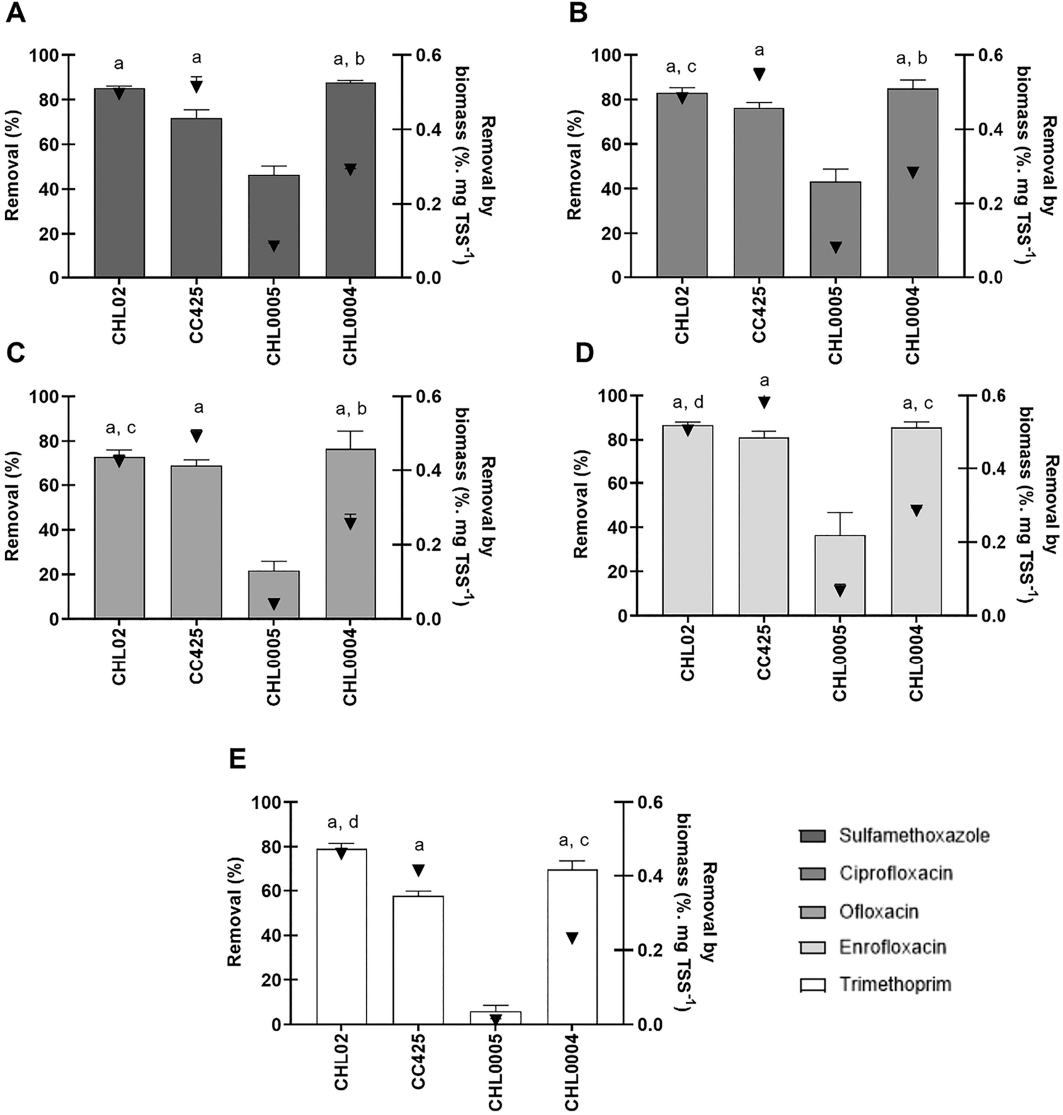
Antibiotic removal (%) and antibiotic removal by biomass (%.mg TSS^–1^) in photobioreactors in a batch aerobic system by *Chlamydomonas* sp. (CHL02 and CC425), *Chlorella* sp. (CHL0005), and *Desmodesmus* sp. (CHL0004) strains. (A) Sulfamethoxazole removal; (B) Ciprofloxacin removal; (C) Ofloxacin removal; (D) Enrofloxacin removal; (E) Trimethoprim removal. Bar graphs represent removal efficiency (%) and symbols (▼) denote removal efficiency per biomass (% mg TSS^–1^). Bars represent standard deviation (*n* = 3). Note: Letters show statistical differences, as per ANOVA. a: Difference with *Chlorella* sp. (CHL0005) (*p* ≤ 0.0001). b: Difference with *Chlamydomonas* sp. strains (CHL02 and CC425) (*p* ≤ 0.0001). c: Difference with *Chlamydomonas* sp. strain (CC425) (*p* ≤ 0.01). d: Difference with *Chlamydomonas* sp. (CC425) and *Desmodesmus* sp. (CHL0004) strains (*p* ≤ 0.008).

Regarding tests with *Chlamydomonas* sp. strains, the one with CHL02 showed mainly sulfamethoxazole and enrofloxacin removal (average removals of 85.11 ± 0.99% and 86.69 ± 1.23%, respectively), whereas that with CC425 showed greater ciprofloxacin and enrofloxacin removal (average removals of 76.24 ± 2.43% and 81.05 ± 2.80%, respectively). Despite better growth and obtaining of biomass, the microbial consortium with *Chlorella* sp. strain achieved statistically lower removal efficiencies for all antibiotics compared to the assays with the other strains (*p* < 0.0001). The best results for that strain regarded removal of sulfamethoxazole (46.47 ± 3.89%) and ciprofloxacin (43.28 ± 5.57%).

ANOVA confirmed the effect of the microbial consortium on the removal of the 5 antibiotics (*F*_(3,8)_ ≥ 1.7 e *p* < 0.0001) by comparing the removal of antibiotics between the strains by biomass (% mg TSS^–1^) (Figure 1). A greater removal efficiency was achieved by *C. reinhardtii* (CC425) (*p* ≤ 0.01) for all antibiotics analyzed, except trimethoprim, whose greatest removal occurred by strain CHL02 (*p* ≤ 0.008). No difference was observed between the two strains regarding removal of sulfamethoxazole (*p* > 0.05). *Chlamydomonas* sp. strain CHL02 was the second most effective in removing antimicrobials, followed by *Desmodesmus* sp.

#### 3.1.2 Tests in the Continuous System

The initial biomass of the consortium’s continuous reactor with Chlorella sp. (CHL0004) was 640.00 ± 9.43 mg L^–1^. Over the 28 days of the continuous reactor operation, a positive result was reported for the removal of the 5 antibiotics (Figure 2)—better results were observed for sulfamethoxazole and trimethoprim, with average removals of 53.73 ± 1.96% and 56.55 ± 4.41%, respectively (*F*_(4,35)_ = 47.41, *p* < 0.0001), with no difference between them. Ciprofloxacin and ofloxacin removals were 34.62 ± 3.44% and 33.81 ± 9.12% respectively, whereas the lowest removal was reported for enrofloxacin, 28.48 ± 4.39%. No statistical differences in their removal were observed.

**Figure 2:**
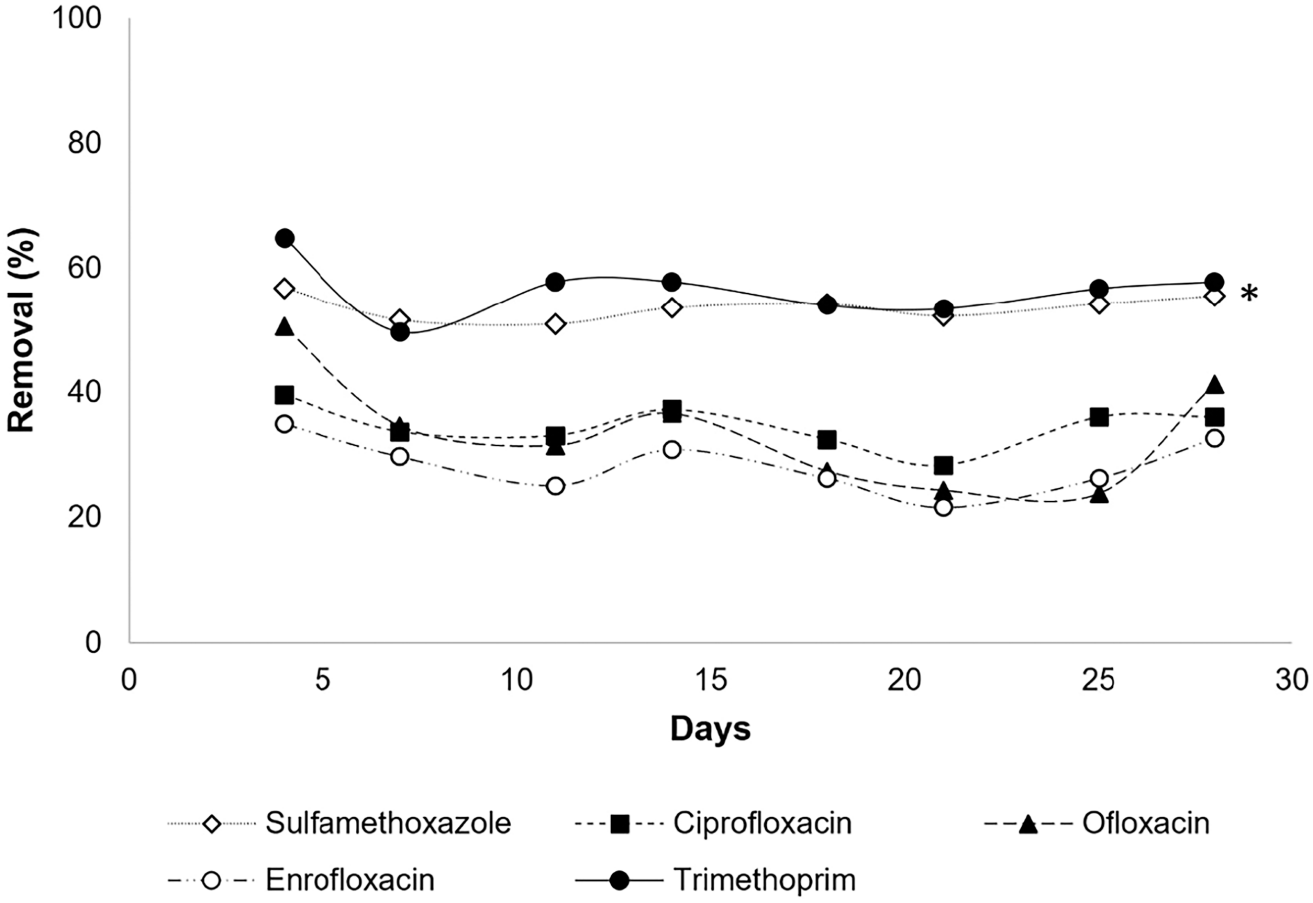
Removal of antibiotics throughout the continuous operation of the reactor system with *Chlorella* sp. strain. * Difference in removal with antibiotics ciprofloxacin, ofloxacin, and enrofloxacin (*p* < 0.0001).

Several studies have demonstrated biological treatment is an effective alternative for the removal of antimicrobials from microalgae and microalgae-bacteria consortium in batch systems and, to a lesser extent, in continuous reactor systems (Table 1). The results of this study have shown and confirmed different potentials of chlorophytes strains, *Chlamydomonas* sp., *Desmodesmus* sp. and *Chlorella* sp., in the five widely used antibiotics from sulfonamide and fluoroquinolone classes. An 81.30 ± 0.98% average removal in the batch system in the test with *Chlamydomonas* sp. (CHL02) and a better removal by biomass by the other strain of *Chlamydomonas* sp. (CC425) (0.51 ± 0.005% mg TSS^–1^) were reported. The continuous reactor system with *Chlorella* sp. showed a 41.44 ± 2.68% average removal.

**Table 1:** Drug Removal by Different Microalgae Strains and by Microalgae-Bacteria Consortium in Batch and Continuous Reactor Systems

	growth conditions										
strain	consortium	growth medium	light or cycle (h)	light int. (umol m^–2^ s^–1^)	*T* (°C)	pH	system	micropollutants (medicines/antibiotics)	assay time (d)	removal rate	
*C. sorokiniana*, *Desmodesmus* sp., *Mesocyclops* sp., and *Parachlorella* sp.	Microalgae -bacteria	Actual secondary effluents	16 h L:8 h D	235 ± 22	22	alkaline	Batch	Sulfamethoxazole	7	54%	^a^
								Trimethoprim Cephalexin		18%	
										97%	
								Erythromycin		92%	
*Chlorella pyrenoidosa*	Axenic condition	TAP medium	16 h L:8 h D	∼70	22		Batch	Triclosan	4	77%	^b^
*C. sorokiniana*	-	Anaerobically treated black	Continuous	68	35	7	Batch Anaerobic	Trimethoprim	31	60%	^c^
*Chlamydomonas* sp.	-	Synthetic wastewater	Continuous	300	20–40	7.2	Batch	Ciprofloxacin Sulfadiazine	9	100 and 54%	^d^
*C. sorokiniana*	-	Standard culture	12 h L:12 h D	370	25 ± 1	7.5	Batch	Paracetamol	8	>67%	^e^
*Chlorella* sp. L38	-	BG-11 m edium	-	∼90	30	-	Batch	Florfenicol	2 0	97%	^f^
*Chlorella* sp. UTEX1602										38%	
*C. reinhardtii* *Dunaliella tertiolecta*	-	TAP m edium	Continuous	172 ± 18	25 ± 1	-	Batch	Sulfapyridine	14	93%	^g^
		Artificial seawater						Ciprofloxacin		100%	
*C. pyrenoidosa*	Axenic conditions	-	-	-	-	-	Batch	Sulfadiazine	12	∼67%	^h^
								Sulfacetam ine		48 ± 3%	
*Chlorella* sp. and *Scenodesmus* sp.	Microbial consortium	Ground water	-	150	23 ± 5	-	Batch	Sulfamethazine	10	62 ± 10%	^i^
								Sulfacetam ide		36 ± 5%	
*Desm odesmus* sp. *C. Pyrenoidosa*	Axenic conditions	TAP m edium	16 h L:8 h D	∼85	25	7.2	Batch	Triclosan	7	100%	^j^
										69.3%	
*Chlorella* sp.	-	Real m unicipal	Continuous	-	24.5 ± 0.5	9.9 ± 0.1	Batch	Hydroxychloroquine	6	89 ± 2%	^k^
*S. almeriensis*	Microalgae-bacteria	Diluted liquid fraction of pig m anure	-	-	-	-	Sem i-continuous	Tetracycline Ciprofloxacin Sulfadiazine	20	77 ± 5%	^l^
										90 ± 14%	
										60 ± 27%	
*C. vulgaris*	Microlagae-bacteria	Piggery wastewater (PWW) -diluted	12 h L:12 h D	1390 ± 30	-	-	Continuous open reactor	Doxycycline Oxytetracycline	92	95 ± 3%	^m^
										93 ± 3%	
*Chlorella* sp. G-9	-	Synthetic wastewater	-	140	28	-	Continuous reactor	Sulfadiazine	63	90 ± 3%	^n^
*Haematococcus pluvialis*	Axenic conditions	Synthetic wastewater	12 h L:12 h D	-	25 ± 1	-	Continuous reactor	Levofloxacin	59	30–57%	^o^
								Flumequine		22–54%	
								Trimethoprim		54–75%	
*Scenedesmus quadricauda*								Levofloxacin		18–46%	
								Flumequine Trimethoprim		15–44%	
										38–66%	

a ref 44.

b ref 45.

c ref 46.

d ref 47.

e ref 48.

f ref 49.

g ref 50.

h ref 51.

i ref 52.

j ref 53.

k ref 54.

l ref 55.

m ref 56.

n ref 57.

o ref 58.

When evaluating the removal efficiency of antibiotics in an aerobic system of secondary effluents from a sewage treatment plant (initial concentration of 50 μg L^–1^ of sulfamethoxazole, trimethoprim, cephalexin, and erythromycin), Rodrigues^44^ observed removals of approximately 54%, 18%, 97%, and 92%, respectively, from the microalgae-bacteria microbial consortium. The consortium showed predominance of *Chlorella sorokiniana* microalgae, despite presence of other genera, such as *Desmodesmus* sp., *Mesocyclops* sp., and *Parachlorella* sp., while *Brevundimonas* sp. and *Rudanella* sp. (Table 1) were the predominant bacteria genera.

Zhou et al.^25^ also demonstrated that a self-suspended algal-bacteria symbiotic system (composed of *Chlorella vulgaris* and *Bacillus subtilis*), using the sol–gel method, enhanced the treatment efficiency of antibiotic-containing wastewater compared to single algal cultures, with 74.2% removal of tetracycline. Another promising approach for the removal of high concentrations of antibiotics is the use of microalgae-bacteria consortium in combination with a gravel matrix, since in wastewater with higher concentrations of these pollutants, the microbial growth and biological functions may be adversely affected.^24^ In this research, polyurethane foam on rods was used as a support material for microbial adhesion in the continuous photobioreactor, in which there was a better result in the removal of 4 of the antibiotics tested compared to the batch system by *Chlorella* sp.

Furthermore, other studies have also demonstrated the removal efficiency of other pharmaceutical compounds from wastewater in anaerobic systems by *C. sorokiniana* (e.g., anti-inflammatory and antimicrobials)^59^ and by *C. acidophila*.^16^ *Chlamydomonas* sp. are chlorophyceae also studied for the removal of ciprofloxacin and sulfadiazine, with 100% and 54% removals, respectively, in batch systems in synthetic wastewater for 9 days.^19^ The present study also showed a 72% ciprofloxacin average removal, with the *Desmodesmus* sp. strain achieving the best removal efficiency for that compound (84.95 ± 3.83%).

A comparison of the results of this research with those of Rodrigues^44^ showed that trimethoprim achieved the lowest removal efficiency in a batch system (Figure 1E), possibly because of its higher difficulty of biodegradation or adsorption. However, a higher trimethoprim removal efficiency was observed in this research, i.e., 4.4 times higher in *Chlamydomonas* sp. The (CHL02) strain was 3.1 times higher with *Chlorella* sp. in the continuous system. Regarding sulfamethoxazole removal, superior results were also achieved (average of 1.3 times higher removal), demonstrating the potential of those strains for micropollutant removal in biological wastewater treatment.

Studies have highlighted the advantages of using microalgae–bacteria consortia for wastewater treatment, as the presence of different organisms utilizing distinct substrates enhances biodegradation rates, enhances nutrient exchange between microorganisms, increases resistance to environmental stressors, and promotes microalgal growth.^24,26^ The mixotrophic metabolism of microalgae allows simultaneous nutrient uptake, which may also be provided by bacteria, and oxygen production via photosynthesis, supporting aerobic degradation of organic carbon by bacteria and thereby enhancing antibiotic removal efficiency in microalgae–bacteria consortia.^22,27,60^ When symbiotic interactions occur between microalgae and bacteria, along with the exchange of metabolites between them, the removal of residual antibiotics may be enhanced through cometabolism, in which the simultaneous and interdependent degradation and transformation of different compounds occur.^22,27^

Other studies have also demonstrated successful removal of micropollutants, such as xenoestrogens, in experiments using the chlorophyte *Ulva mutabilis* in consortium with the bacteria *Roseovarius* sp. and *Maribacter* sp., in which removal occurred mainly through biodegradation. However, contrary to findings from other studies highlighting the advantages of algae–bacteria consortia in micropollutant removal, the researchers observed removal rates higher than 99% for bisphenols A, B, E, F, P, and Z, and partial removal of bisphenol S under axenic conditions, indicating that bacteria did not influence the biodegradation process. Nevertheless, chlorophyte may support bacterial growth by creating protective microenvironments that shield bacteria from harmful conditions in aquatic ecosystems.^61^

In this context, different microalgae–bacteria or algae–bacteria consortia may exhibit distinct behaviors and mechanisms in the removal of different types of micronutrients, highlighting the need for more in-depth investigations. Microorganisms possess different mechanisms for antibiotic removal, such as biosorption, bioaccumulation, and biodegradation. Photodegradation, volatilization, and hydrolysis may also occur; however, they contribute to a lesser extent to the overall removal process.^7,13^ These wild strains, in this initial investigation, may have employed different mechanisms for antibiotic removal, and will be the subject of future investigation.

However, several studies have demonstrated that biosorption is the main mechanism used to remove antibiotics such as metronidazole and ciprofloxacin by *Chlorella* ^62,63^ and tetracycline, ciprofloxacin, sulfadiazine, and sulfamethoxazole by *S. almeriensis*.^22^ *Chlamydomonas* has demonstrated positive results in the removal of sulfamethoxazole and ciprofloxacin, also used in this research, via biodegradation and bioadsorption.^19^ Furthermore, the results of the abiotic control in this study (SI C), as well as those reported in other studies, indicate that the removal of antibiotics through hydrolysis, photolysis, adsorption, and volatilization is negligible.^13,22,23^

### 3.2 Nutrients Removal

#### 3.2.1 Batch System Tests

In addition to antimicrobials removal, tests with microbial consortia also removed macronutrients—nitrogen, phosphorus, and organic carbon (Table 2). ANOVA confirmed the effect of the strain on nutrient removal (F _(3;8)_ ≥ 66.5 and *p* < 0.0001). A comparison of the strains revealed organic carbon and nitrogen were better removed in tests with *C. reinhardtii* (CC425), whereas *Desmodesmus* sp. (CHL0005) was the most efficient for phosphorus removal.

**Table 2:** Percentage of Removal of Macronutrients by the Microbial Consortium of the Four Strains and Removal by Biomass (% mg TSS^–1^) for Comparisons of Their Efficiency^a^,^b^,^c^,^d^,^e^

			organic carbon	
strain	nitrogen	phosphor	COD	carbohydrate
	removal (%)			
*Chlorella* sp.	74.4 ± 6.0	23.5 ± 4.2	52.6 ± 7.4	66.0 ± 9.6
*Desmodesmus* sp.	75.3 ± 6.9	90.9 ± 5.9	80.9 ± 5.9	88.6 ± 0.5
*Chlamydomonas* sp. (CHL02)	34.0 ± 15.8	0	83.2 ± 1.2	85.9 ± 2.2
*C. reinhardtii* *(*CC425)	89.0 ± 1.1	2.9 ± 3.1	88.7 ± 6.0	94.5 ± 0.6
	nutrient removal by biomass (% mg TSS^–1^)			
*Chlorella* sp.	0.14 ± 0.01	0.04 ± 0.01^a^	0.10 ± 0.01	0.12 ± 0.02
*Desmodesmus* sp.	0.25 ± 0.02	0.30 ± 0.02^a^	0.27 ± 0.02^a^	0.30 ± 0.00^a^
*Chlamydomonas* sp. (CHL02)	0.20 ± 0.09	0	0.49 ± 0.01^a^	0.50 ± 0.01^a^
*C. reinhardtii* (CC425)	0.64 ± 0.01^a^	0.02 ± 0.02	0.64 ± 0.04^a^	0.68 ± 0.00^a^

a Letters indicate statistical differences according to ANOVA.

b Difference with all strains (*p* ≤ 0.0003).

c Difference with *Chlamydomonas sp*. strain (CHL02) (*p* = 0.034).

d Difference with *Desmodesmus sp*. and Chlorella sp. strains (*p* ≤ 0.0001).

e Difference with *Chlorella sp*. strain (*p* ≤ 0.0001).

Although incapable of removing phosphorus, the *Chlamydomonas* sp. (CHL02) strain showed positive results in the test mainly for wastewater rich in organic carbon treatment, with the second best result for that parameter. *Chlorella* sp. (CHL0004) was the second most efficient for phosphorus removal (0.043 ± 0.008% mg TSS^–1^); however, it achieved the lowest organic carbon removal efficiency.

Kumar et al.^64^ conducted experiments with phosphorus and obtained a 55% average removal using *Chlorella* sp., and Sarfraz et al.^65^ achieved a 71% removal with *Desmodesmus subspicatus* in their tests. The present study obtained a 1.28 times higher efficiency (90.9%) in the microbial consortium with a strain of the same genus. Regarding total nitrogen removal, greater emphasis was placed on the microbial consortium with *Chlorella* sp. (74.4%) and *Desmodesmus* sp. (75.3%) strains, as in Kumar et al.,^64^ who reported a 91% average removal with *Chlorella* sp.

This study has shown that other strains of chlorophyte’s tests have yielded better results compared to *Chlorella* sp. Bolani et al.^28^ also proved that *Desmodesmus* sp. is more efficient in biological treatment wastewater compared to *Chlorella* sp. in batch bioreactors under various light regimes. However, other researchers stated strains of *Chlorella* sp. genus are efficient in the bioremediation of macronutrients and micronutrients, such as carbon dioxide, phosphates, sulfur dioxide, nitrogen, among others.^14,26^ Chaudhary et al.^66^ reported the result for macronutrients bioremediation in batch photobioreactors was also overwhelming for *C. vulgaris*, which removed 74.4% of organic matter, measured by COD, similarly to the present results, as well as 81.93% phosphorus removal.

In a wastewater bioremediation study, Kumar et al.^64^ obtained a 75% removal efficiency of organic carbon using *Chlorella* sp. with photoperiod for 9 days. Using *D. subspicatus*, Sarfraz et al.^65^ reported an 84% COD removal under 24 h light conditions, whereas Ogbonna et al.^67^ observed 89% removal in 24 h in the dark. The present results with *Desmodesmus* sp. in relation to organic carbon removal were similar but more satisfactory with *Chlamydomonas* sp. strains.

Other studies, including the present one, evaluated the removal of macronutrients alongside that of antibiotic removal. Pereira et al.^11^ reported that the physicochemical characteristics of wastewater influenced pharmaceutical removal, with significant positive correlations observed between the concentration of total nitrogen and the removal efficiency of drugs.

The relationship between nutrient and antibiotic removal in biological systems, such as microalgae–bacteria consortia, is mainly attributed to metabolic and biochemical processes that affect both nutrient and micropollutant removal due to the cometabolism that can occur within these microbial consortia. The exchange of metabolites between microalgae and bacteria can lead to the cometabolic degradation of antibiotics and nutrient removal, potentially making these systems more efficient for micropollutant elimination.^22,27^ Moreover, an environment with sufficient nutrients maintains microbial activity, promotes growth, and increases biomass production, which can consequently enhance antibiotic removal.^24,60^

The removal efficiencies of organic carbon may not be so evident due to the generation of organic acids. All tests generated byproducts with potential industrial applicability. *Chlamydomonas* strains produced the least organic acids, with CC425 strain generating propionic acid (7.75 ± 1.54 mg L^–1^) and CHL02 strain producing isobutyric acid (5.79 mg L^–1^). The assay with *Chlorella* sp. generated formic, acetic, and lactic acids (6.15 ± 3.17, 69.70 ± 5.89, and 4.71 ± 2.50 mg L^–1^, respectively), whereas *Desmodesmus* sp. produced acetic (12.52 ± 3.80 mg L^–1^) and lactic (12.93 ± 3.58 mg L^–1^) acids.

#### 3.2.2 Continuous System Test

Average organic carbon removal efficiencies of 96.64% ± 1.67% and 67.75% ± 15.27% were achieved for carbohydrate and COD, respectively, 58.99% ± 7.74% for nitrogen, and 41.63% ± 15.21% for phosphorus in the test with *Chlorella* sp. in a continuous reactor (Figure 3). A statistically significant difference was observed among nutrient removal efficiencies during the operation of the continuous reactor (*F*_(3,28)_ = 25.98, *p* < 0.0001), with carbohydrate removal being significantly higher than that of all other nutrients, and COD removal exceeding that of phosphorus. Except for nitrogen, the results evidenced the removal of all nutrients in a continuous system by the microbial consortium formed by *Chlorella* sp. was more efficient than that in the batch system, especially regarding carbohydrate removal, with 1.5 times greater efficiency.

**Figure 3:**
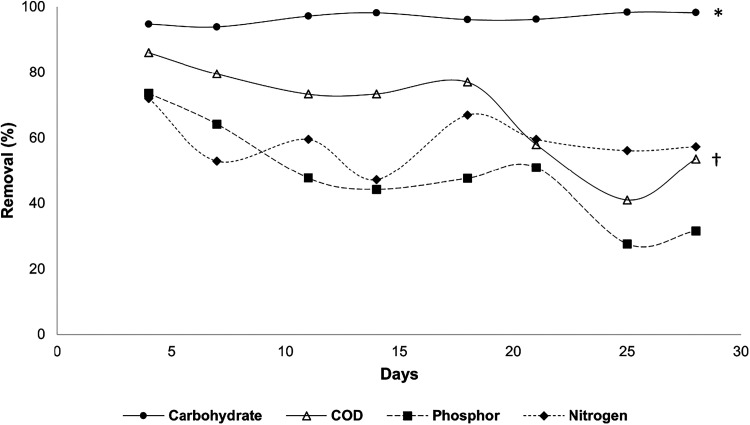
Removal of macronutrients throughout the operation of the continuous reactor system with *Chlorella* sp. strain. * Difference in removal with nitrogen, phosphor, and COD (*p* ≤ 0.0001). † Difference in removal with phosphor (*p* = 0.01).

Other studies with *Chlorella* sp. also obtained significant results for nutrient removal. In tests conducted in an open environment with those microalgae, Moondra et al.^68^ achieved a 78% average removal efficiency of COD, approximately 87% of inorganic phosphorus, and 86% of nitrogen. When analyzing the removal of COD and total phosphorus by *C. vulgaris* grown in a mixed culture medium and on a laboratory bench scale, Neto et al.^69^ obtained the best removal efficiencies of around 70.57% and 97.96%, respectively. Kumar et al.^64^ reported a lower result of COD removal in a continuous system, reaching 42% with *C. vulgaris*, despite 52% and 83% removals of phosphorus and nitrogen, respectively, with the same chlorophyte in wastewater.

Therefore, as observed by Kumar et al.,^64^ the factors that influence a lower COD removal efficiency are the daily nutrients/influents supply. However, the lower COD removal can also be influenced by the release of organic compounds produced by chlorophyte species in their metabolism.^70^ As performed in this research, the presence of organic acids produced and released into the medium by chlorophyceae must be taken into account during the verification of the efficiency of this strain in removing total carbon by COD. Analyses of carbohydrate removal are therefore useful for determining the capacity of microalgae for removing organic carbon.

Regarding the byproducts generated in the continuous reactor with *Chlorella* sp., isobutyric, acetic, and butyric acids (averages of 10.85, 85.99, and 47.32 mg L^–1^, respectively) were produced throughout the reactor’s operation, although the production of the latter two was larger in the last 8 days of operation (158.21 and 72.19 mg L^–1^, respectively). Propionic and isovaleric acids (10.17 and 16.21 mg L^–1^, respectively) were produced at the end of the reactor operation.

After 28 days of reactor operation, gas production was also detected, with a total volume of 3.69 mL of CO_2_ and 0.05 mL of H_2_. The hydrogen generated resulted from the hydrolysis of water and the breakdown of organic carbons by the microalgae in a microbial consortium and can be used as a biofuel.^71^ Although the total volume of hydrogen produced and maintained in the bag was low (0.05 mL) (13.39 μmol L^–1^), that strain can potentially generate hydrogen, thus requiring further investigations toward optimizing both process and production of that biogas.

Due to the metabolic diversity of mixotrophic,^71^ studies have demonstrated its potential to generate alcohols, organic acids, and biogas. Vargas et al.^10^ reported that three different strains of *Chlamydomonas* sp. produced mainly hydrogen, alcohols, and formic, lactic, isobutyric, and acetic acids in wastewater with different organic carbon sources. However, unlike the conditions of this research, which may justify the lower yields of organic acids and the absence of alcohol production, anaerobic conditions have promoted positive results and higher byproduct yields due to fermentation pathways.^12,71^

### 3.3 Batch System versus Continuous System with *Chlorella* sp

A comparison of the removal of antimicrobials between discontinuous and continuous systems by the microbial consortium in tests with *Chlorella* sp. strain revealed a statistically higher removal of sulfamethoxazole and trimethoprim in the continuous system (*p* ≤ 0.0021), i.e., approximately 1.2 and 9.5 times higher compared to the batch system. No differences were detected in the removal of enrofloxacin and ofloxacin between the systems, although the average removal of the latter was 1.6 times greater in the continuous reactor (*p* = 0.059). Ciprofloxacin was the only antimicrobial that statistically obtained the best removal result in the batch system (1.25 times greater, *p* = 0.01).

Among the most desired characteristics of microalgae for use in biological treatments are higher growth rates, possible resistance to pollutants, and environmental variations.^72^ *Chlorella* sp. has been widely used for it is tolerant to external factors, can easily adapt to different culture media, and, consequently, has intensified and accelerated growth, favoring its use for bioremediation purposes.^68,69^

The aforementioned points became clear during the ecotoxicological test with *Chlorella* sp. (SI B), which showed better growth in synthetic wastewater, and, therefore, was selected for the antimicrobial removal in a continuous system reactor. The results also proved that the chlorophyte strain, on a larger scale, generally shows better removal efficiency compared to the discontinuous system, except for ciprofloxacin. Studies have demonstrated the potential efficiency of *Chlorella* sp. in removing drugs (see Table 1). Furthermore, the tests conducted in this investigation revealed that the *Chlorella* sp. strain removed not only antimicrobials but also almost 100% of organic carbon in a continuous system.

The difference in antimicrobial removal between batch and continuous systems can be attributed to the suspension of cells in the first system and the partial immobilization of the microbial community on the polyurethane foam support in the continuous system. The findings of this study are consistent with previous reports in the literature, which indicate that immobilized cells or the use of support materials leads to higher removal rates.^24,25^ Furthermore, the different systems can also influence the predominant mechanisms of antibiotic removal, which occur in different proportions through bioadsorption, bioaccumulation, and biodegradation.^7,73^

Some research has introduced innovations to further enhance the efficiency of biological treatment of antibiotics with microalgae, such as optimizing environmental and operational conditions, employing hybrid systems that combine microalgal treatment with conventional activated sludge, membrane filtration, or pretreatment with physical and chemical processes, as well as applying genetic engineering and omics technologies to deepen the understanding of removal mechanisms.^73,74^ These strategies can serve as the focus of future investigations involving these promising wild Brazilian strains, which have shown an effective removal of widely used antibiotics. Another promising biological alternative that has been investigated for micropollutant removal is the model system *Ulva*. This marine green alga has been widely suggested for use as a biofilter for bioremediation in integrated multitrophic aquaculture due to its resilience to many micropollutants.^61^

Another concern is the presence of antibiotic resistance genes (ARGs), which may arise as a response to antibiotic-induced stress in microalgae–bacteria consortia and are frequently disseminated in wastewater treatment plants through horizontal gene transfer. Microalgae–bacteria consortia can remove antibiotics and consequently reduce the abundance of these genes; however, environmental factors such as metals, nutrients, pH, and temperature may influence their persistence and dissemination.^27,75,76^

Therefore, since this preliminary study detected positive antibiotic removal results using these chlorophyte strains in consortia with bacteria never previously studied for this purpose, it may contribute to future investigations addressing the gaps observed here, particularly regarding the elucidation of the different removal mechanisms of these compounds given the presence of complex mixtures of various antibiotics at varying concentrations in real wastewater. Furthermore, it highlights the need for more in-depth studies on cometabolism involving microbial consortia formed by these strains as well as the development of technologies that enable the economic viability and large-scale application of these microorganisms in the biological treatment of wastewater.

*Chlorella* is a promising microalgae genus for use in biological treatment aimed at the removal of micropollutants commonly found in aquatic ecosystems. However, it is worth noting that even higher antibiotic removal efficiencies were observed in this study in batch system assays with *Chlamydomonas* sp. and *Desmodesmus* sp., which warrants further investigation at larger scales and using real wastewater with these potential strains in microalgae–bacteria consortia.

## 4 Conclusions

Four wild strains of microalgae isolated in Brazil not previously studied, in consortium with autochthonous bacteria in photobioreactors in a batch system, were efficient in the removal of antibiotics and macronutrients. In tests with strains of *Chlamydomonas* sp., (CHL02) and *Desmodesmus* sp., the best results were achieved for the removal of the 5 antibiotics tested, with 81% average removal. However, *C. reinhardtii* (CC425) showed the best average biomass removal efficiency (0.51% mg TSS^–1^). The test with the *C. reinhardtii* (CC425) strain was the most efficient for the removal of organic carbon and total nitrogen, and the one with *Desmodesmus* sp. provided the best result for the removal of inorganic phosphorus. In the continuous reactor system, the microbial consortium with *Chlorella* sp. achieved 97% removal of organic carbon, along with higher removal efficiencies for sulfamethoxazole (54%) and trimethoprim (57%). This genus showed a higher removal efficiency for most of the tested antibiotics in the continuous system compared to the batch system. Therefore, the preliminary results from all natural chlorophyte consortia investigated in this study indicate that they are effective alternatives for the removal of nutrients and antibiotics in biological treatment of urban wastewater. However, more in-depth investigations are required to elucidate the mechanisms of antibiotic removal by microorganisms and the interactions that are established between them.

## Supplementary Material



## Notes

Description of the synthetic wastewater used in the study (SI A), the preliminary results of the ecotoxicological assays (SI B), abiotic control to evaluate the removal of the antibiotics (SI C), and the parameters applied in the HPLC-MS/MS analysis of antibiotics (SI D) (PDF)
