# Supplementary material for: Different Strains of Microalgae in a Microbial Consortium Remove Antibiotics from Urban Wastewater in Batch and Continuous Treatment Systems
Source: ACS Omega. 2026 Apr 30;11(18):27635–47. doi: 10.1021/acsomega.6c02775 (PMC13177219; doi:10.1021/acsomega.6c02775)

Different strains of microalgae in a microbial consortium remove antibiotics from urban wastewater in batch and continuous treatment systems

Sarah Regina Vargas[a, b, *], Julia de Lima Santos[a], Pedro Henrique Ferraz Ribeiro[a], Iliseu Monteiro Alcântara[a], Rodrigo Braz Carneiro[c], Marcelo Zaiat[b]

[a] Laboratory of Microbiological Processes, Department of Biomedical Sciences and Health, Minas Gerais State University (UEMG), 164, Sabará St., Centro, Passos, Minas Gerais, 37900-004, Brazil.

[b] Laboratory of Biological Processes, São Carlos School of Engineering, University of São Paulo, 1100, João Dagnone Ave., Santa Angelina, São Carlos, São Paulo, 13563-120, Brazil.

[c] Laboratory of Chromatography, São Carlos Institute of Chemistry, University of São Paulo (USP), 400, Trabalhador São-Carlense Ave., São Carlos, São Paulo 13566-590, Brazil.

* Corresponding author: sarah.vargas@uemg.br

Julia de Lima Santos: Laboratory of Human Cytogenetics and Cytogenomics. Department of Medical Genetics and Genomic Medicine, University of Campinas. s/n., Albert Sabin St, Zip Cidade Universitária. Zeferino Vaz, Campinas, São Paulo, 13083-894, Brazil.

Supporting Information

SI A - Synthetic wastewater proposed by (Torres, 1992) adapted by (Carneiro et al., 2019).

Table S1. Components of synthetic wastewater

**Components**	**Concentration**
Sucrose	211 mg L^{-1}
Starch	221 mg L^{-1}
Meat extract	530 mg L^{-1}
Sodium bicarbonate ($NaHCO_3$)	600 mg L^{-1}
Salt solution (A)	2 mL L^{-1}
Phosphorus solution (B)	1 mL L^{-1}
Micronutrients solution (C)	1 mL L^{-1}

Table S2. Components of salt solution (A)

**Components**	**Concentration** mg L^{-1}
Sodium chloride (NaCl)	250
Magnesium chloride ($MgCl_2.6H_2$)	28
Calcium chloride ($CaCl_2.2H_2O$)	18

Table S3. Components of phosphorus solution (B)

**Components**	**Concentration** mg L^{-1}
Potassium phosphate monobasic (KH_2PO_4)	30

Table S4. Components of micronutrients solution (C)

Components	Concentration mg L^{-1}
Nitrilotriacetic Acid (NTA)	12.8
Ferric chloride hexahydrate ($FeCl_3.6H_2O$)	1.35
Manganese chloride tetrahydrate ($MnCl_2.4H_2O$)	0.1
Cobalt chloride hexahydrate ($CoCl_2.6H_2O$)	0.024
Calcium chloride dihydrate ($CaCl_2.2H_2O$)	0.1
Zinc chloride tetrahydrate ($ZnCl_2.4H_2O$)	0.1
Cupric chloride tetrahydrate ($CuCl_2.4H_2O$)	0.025
Boric acid (H_3BO_3)	0.01
Sodium molybdate dihydrate ($Na_2MoO_4.2H_2O$)	0.024
Sodium chloride ($NaCl$)	1.0
Sodium selenium pentahydrate ($Na_2SeO_3.5H_2O$)	0.026
Nickel chloride hexahydrate ($NiCl_2.6H_2O$)	0.12

SI B - Preliminary results of ecotoxicological testing

The strains' growth was monitored for 10 days by absorbance (680 nm) and no inhibition was observed under the tested conditions with 100 μg L^{-1} in synthetic wastewater, compared to control condition (antibiotic-free wastewater, containing only methanol).

The *Chlorella vulgaris* strain (CHL0005) showed growth inhibition in synthetic wastewater only at a concentration of 100 mg L^{-1} with the antibiotics enrofloxacin and ofloxacin, and better growth at a concentration of 0.1 mg L^{-1} with all the antibiotics tested (Figure S1).

The *Desmodesmus* sp. strain (CHL0004) in synthetic wastewater with all the antibiotics at a concentration of 0.1 mg L^{-1} did not cause growth inhibition. The same happened at a concentration of 10 mg L^{-1} of the antibiotics ofloxacin and enrofloxacin. Only sulfamethoxazole did not interfere with the strain's growth at any of the concentrations (Figure S2).

For the *Chlamydomonas* sp. strain (CHL02), there was no growth inhibition with the antibiotics trimethoprim, ciprofloxacin and sulfamethoxazole at all the concentrations tested, except for the condition with 100 mg L^{-1} of ciprofloxacin. In relation to the antibiotics ofloxacin and enrofloxacin, there was less growth (Figure S3).

In the test with the *Chlamydomonas reinhardtii* strain (CC425), the lowest concentrations of antibiotics did not generate growth inhibition. Growth inhibition occurred at the highest concentration with all the antibiotics and there was no growth with ofloxacin (Figure S4).

Figure S1. Ecotoxicological test with the *Chlorella* sp. (CHL0005) strain in synthetic wastewater.

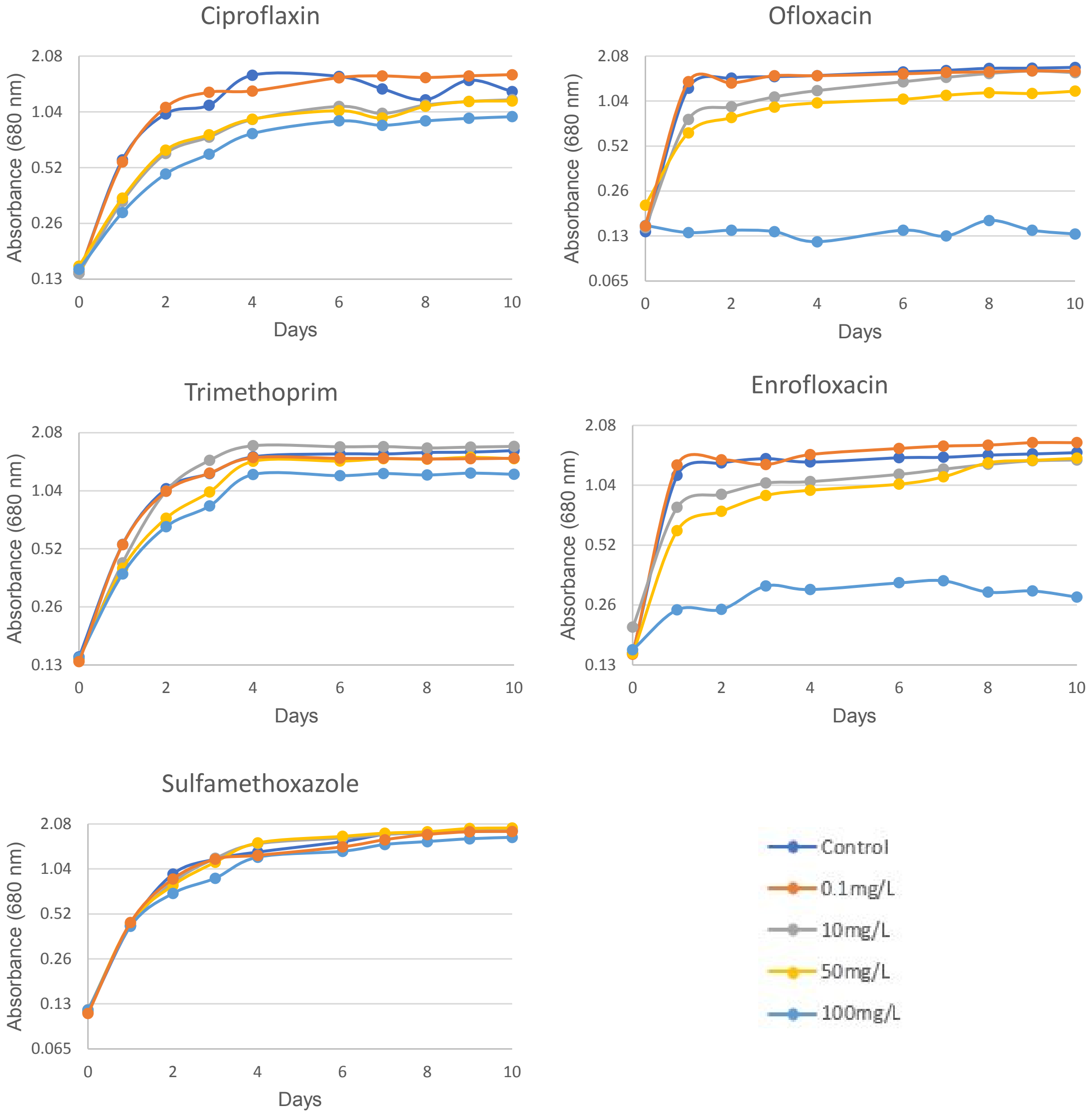

Figure S2. Ecotoxicological test with the *Desmodesmus* sp. (CHL0004) strain in synthetic wastewater.

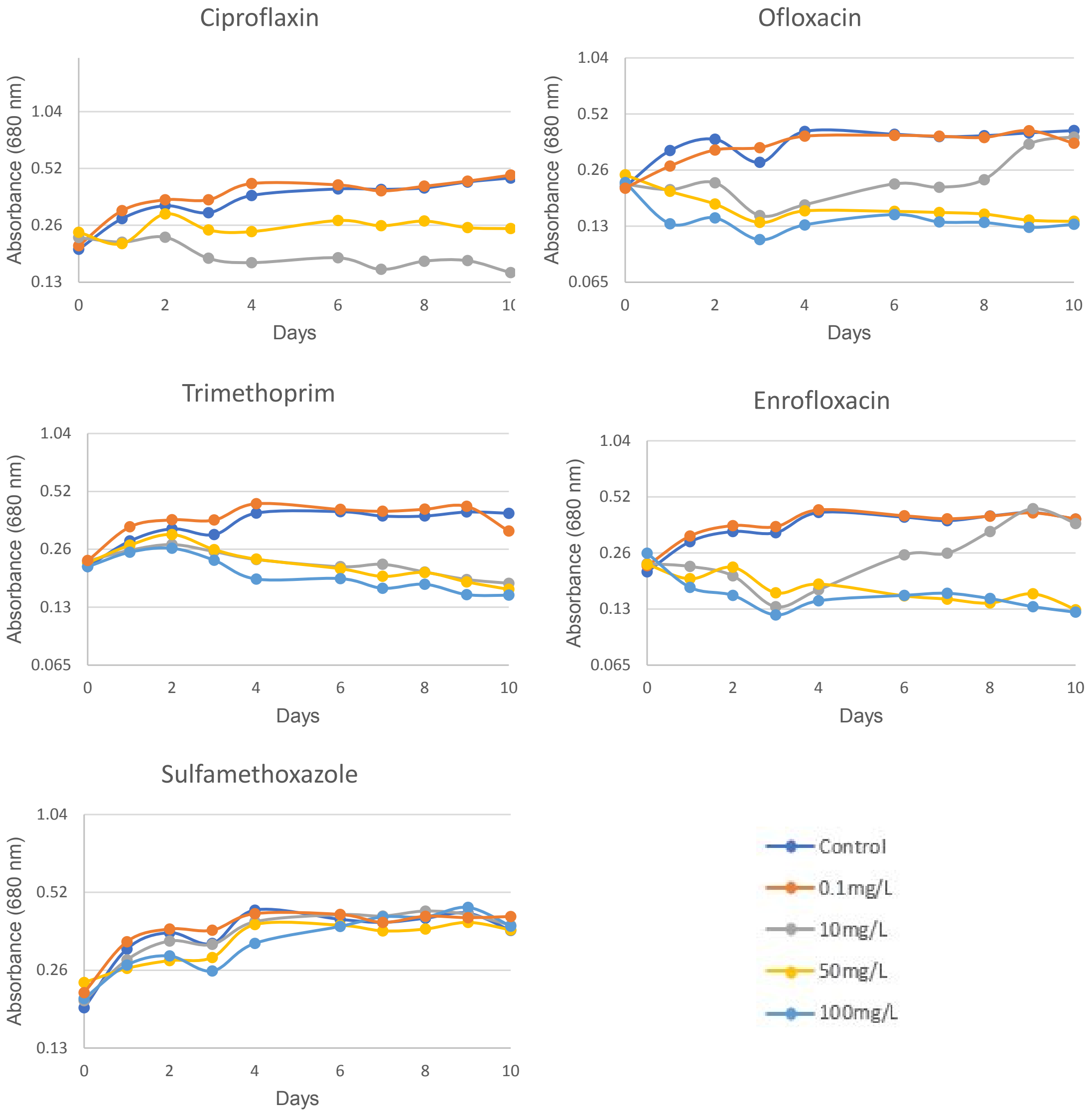

Figure S3. Ecotoxicological test with the *Chlamydomonas* sp. strain (CHL02) in synthetic wastewater.

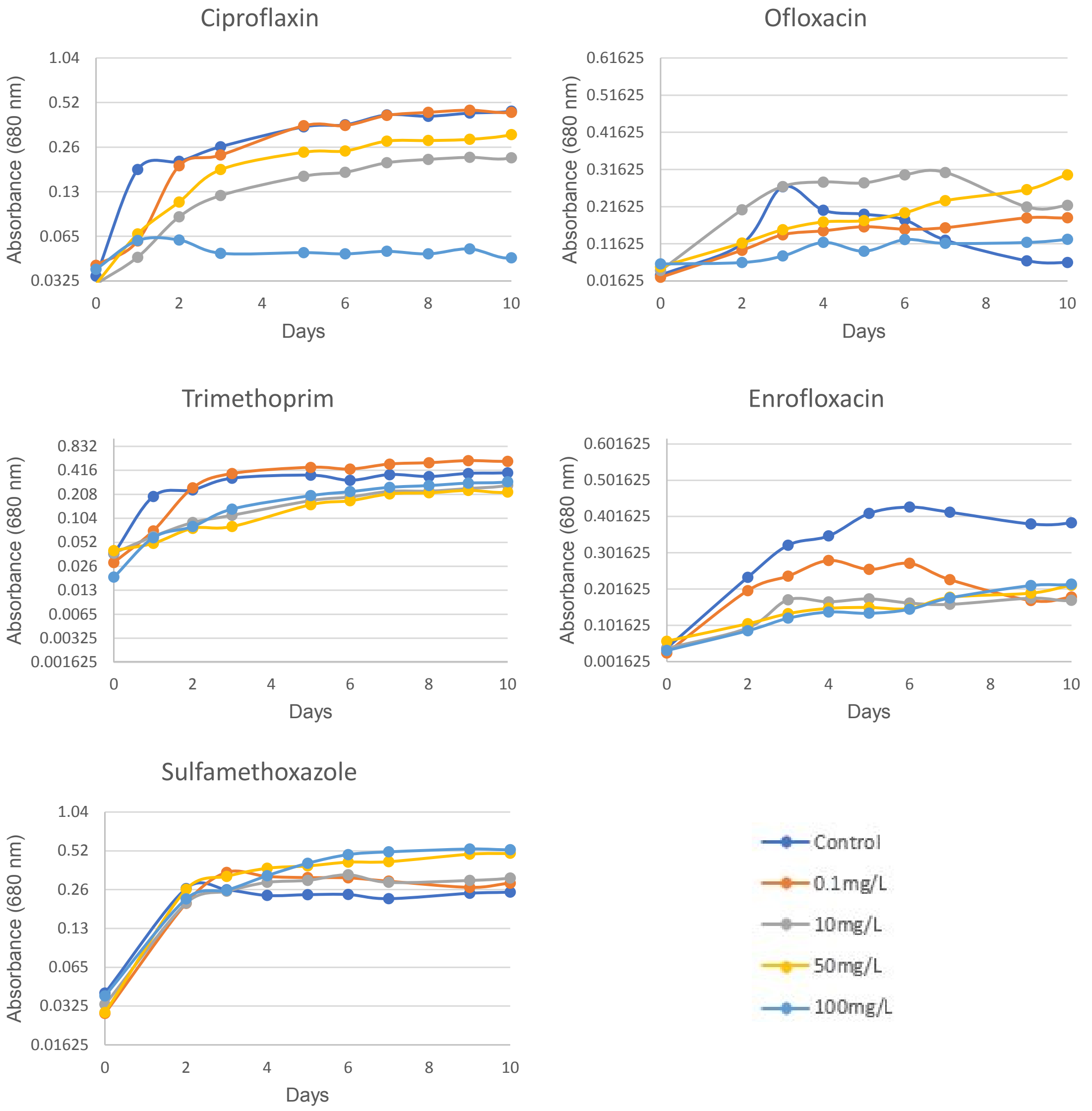

Figure S4. Ecotoxicological test with the *Chlamydomonas reinhardtii* strain (CC425) in synthetic wastewater.

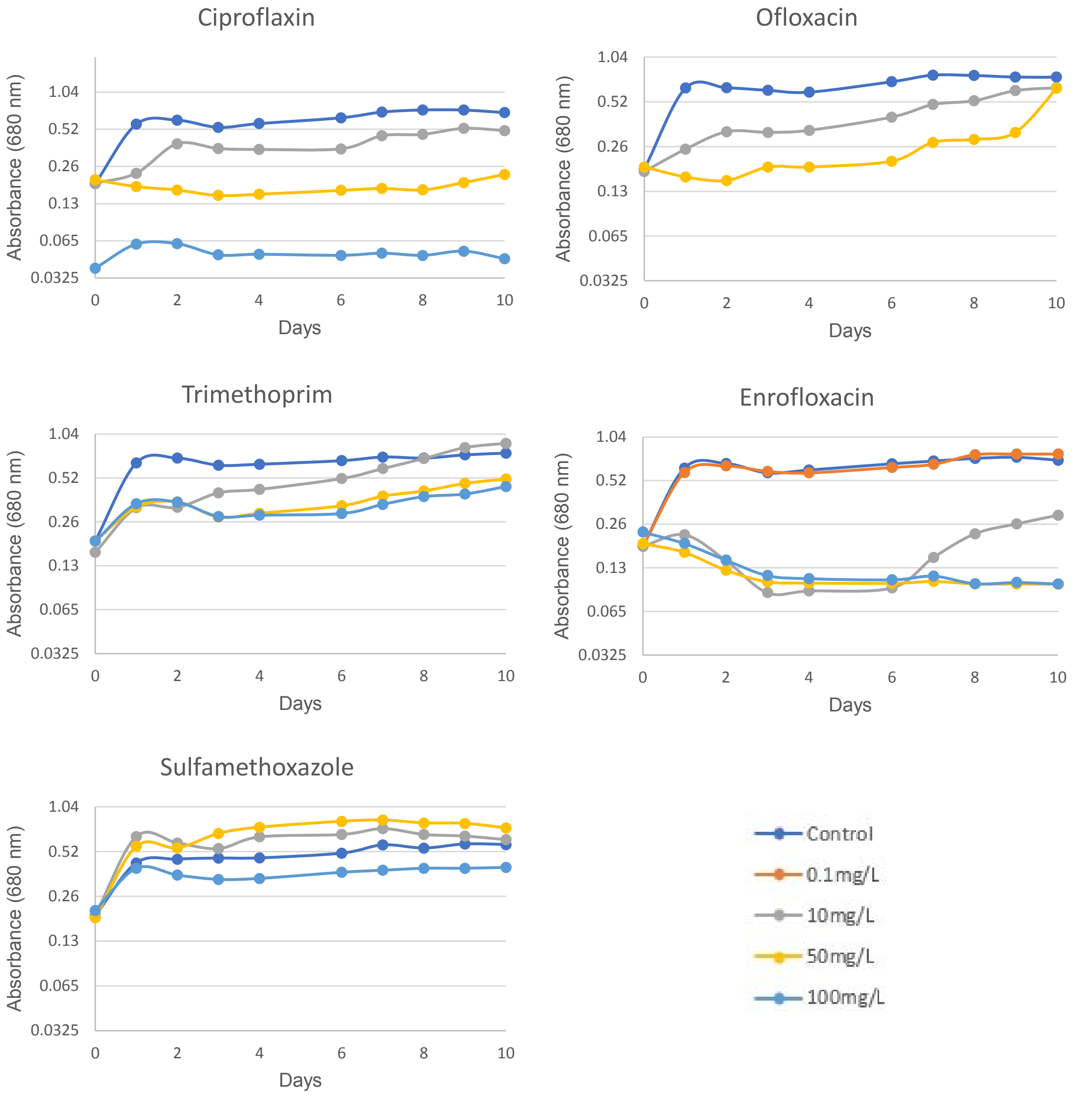

The results of the EC_{50} analysis (Figure S5) indicated that *Chlorella* sp. (CHL0005) was the most resistant strain to antibiotics, particularly to sulfamethoxazole, ofloxacin, and enrofloxacin (EC_{50} > 100 mg L^{-1}), while it was more sensitive to ciprofloxacin (EC_{50} = 3.59 mg L^{-1}). Considering this result, an evaluation of the data behavior (Hormesis effect) was performed.

CHL02 exhibited greater sensitivity to ofloxacin (EC_{50} = 0.06 mg L^{-1}) and to trimethoprim (EC_{50} = 3.20 mg L^{-1}), whereas *Chlamydomonas* sp. (CC425) and *Desmodesmus* sp. (CHL0004) showed higher sensitivity to enrofloxacin (EC_{50} = 1.03 mg L^{-1}) and ciprofloxacin (EC_{50} = 1.08 mg L^{-1}), respectively, although CHL0004 also showed sensitivity to trimethoprim, ofloxacin and enrofloxacin antibiotics (EC_{50} ≤ 3.99 mg L^{-1}). CC425 and CHL0004 were more resistant to sulfamethoxazole (EC_{50} ≥ 102.13 mg L^{-1}) and to ciprofloxacin by CC425 (EC_{50} = 153.42 mg L^{-1}).

Figure S5. EC_{50} calculations of the five antibiotics (sulfamethoxazole, trimethoprim, ofloxacin, ciprofloxacin, and enrofloxacin), at four different concentrations (0.1, 10, 50 and 100 mg L^{-1}) with *Chlorella* sp. and the Brain-Cousens hormesis models for *Chlorella* sp., *Desmodesmus* sp., and two strains of *Chlamydomonas* sp. (CHL02) and *Chlamydomonas* sp. (CC425).

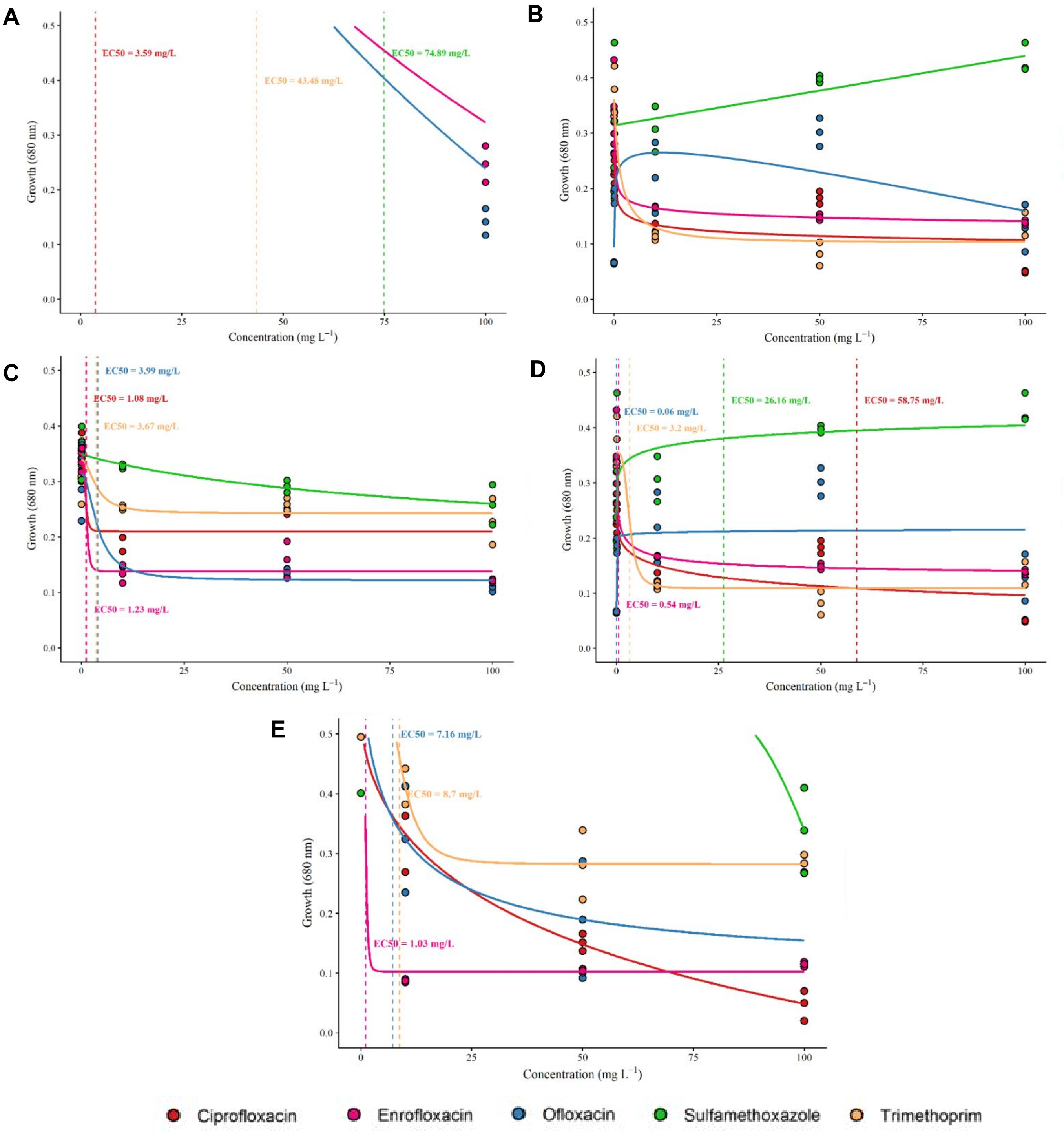

Chlorella sp. (CHL0005) (A) and the Brain-Cousens hormesis models (B), *Desmodesmus* sp. (CHL0004) (C), and two strains of *Chlamydomonas* sp. (CHL02) (D) and *Chlamydomonas* sp. (CC425) (E).

SI C - Abiotic control to evaluate the removal of the antibiotics

Figure S6. Abiotic control for the removal of the antibiotics sulfamethoxazole, trimethoprim, ofloxacin, ciprofloxacin, and enrofloxacin, showing the initial and final concentrations under light conditions and in the dark.

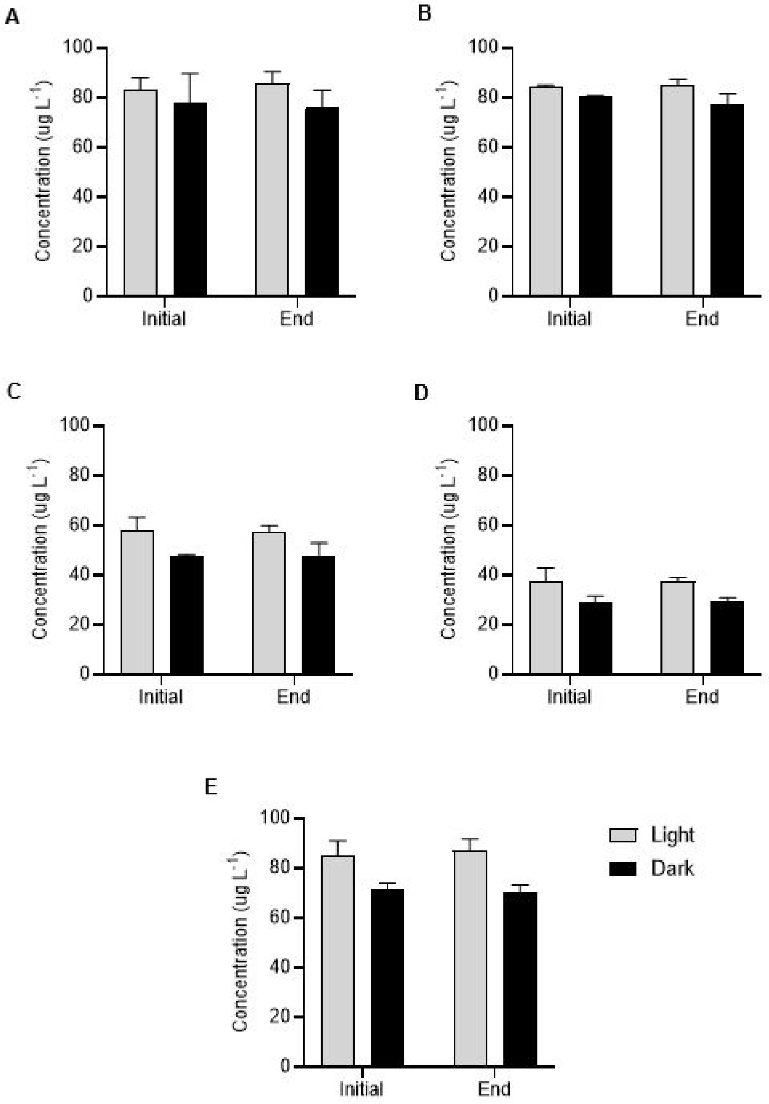

The graphs represent the abiotic controls for antibiotic removal: sulfamethoxazole (A), trimethoprim (B), ofloxacin (C), ciprofloxacin (D), and enrofloxacin (E). No statistically significant difference was observed between the initial and final concentrations of all antibiotics, under both light and dark conditions.

SI D - Parameters used in HPLC-MS/MS for antibiotics analysis

For analysis of the concentrations of antibiotics was used a liquid chromatograph (Acquity UPLC model, consisting of a binary pump, automatic sampler and column oven) coupled to a triple quadrupole mass spectrometer (XEVO TQ MS model) of the Waters Corporation.

The column used was the Poroshell 120 EC-C18 3.0 x 50mm 2.7 µm (Agilent). The aqueous mobile phase (A) consisted of ultrapure water containing formic acid 0.1% (v/v), and

as an organic mobile phase (B) a solution of acetonitrile containing formic acid 0.1% (v/v) was applied, with a flow rate of 0.35 mL min^{-1}. The column and injector temperatures were 30 ºC and 20 °C, respectively, and the injected sample volume was 3 µL. The mass spectrometer conditions were:

- Source: electrospray (positive mode)
- Capillary voltage: 3 kV
- Desolvation gas temperature (500 ºC)
- Desolvation gas flow (1000 L/h)
- Ion source temperature: 150 ºC
- Monitored transitions: Table 1

Table S5. Monitored transitions of the compounds

COMPOUND	TRANSITION	CONE ENERGY (V)	COLLISION ENERGY (V)	DWELL TIME (s)
Sulfamethoxazole	254 > 108*	30	25	0.02
Sulfamethoxazole	254 > 156	30	15	0.02
Trimethoprim	291 > 123*	30	30	0.02
Trimethoprim	291 > 230	30	25	0.02
Ciprofloxacin	332 > 314*	20	20	0.02
Ciprofloxacin	332 > 288	20	20	0.02
Enrofloxacin	360 > 342*	15	15	0.02
Enrofloxacin	360 > 316	15	15	0.02
Ofloxacin	362 > 261*	20	30	0.02
Ofloxacin	362 > 318	20	20	0.02

* Quantification transition

Figures S7 and S8 show the representation of the illustrative chromatograms with the compounds together and separately, respectively, at a concentration of 50 µg L^{-1}.

Figure S7. Illustrative chromatogram – analyte concentration of 50 µg L^{-1}.

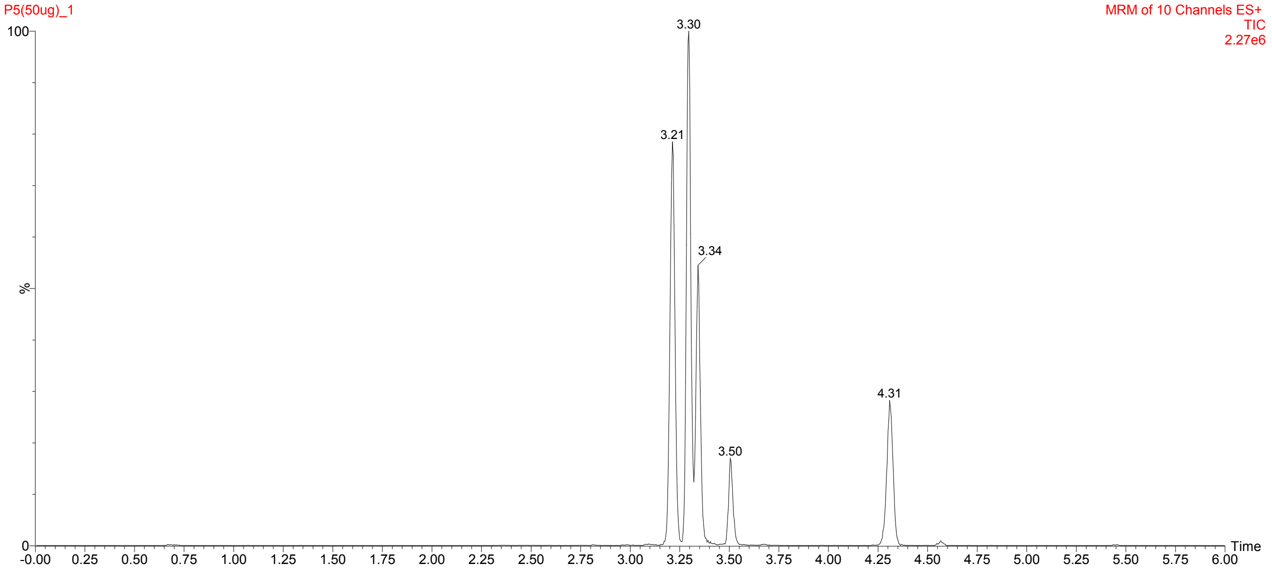

Figure S8. Illustrative chromatogram (separate MRM transitions) – analyte concentration of 50 µg L^{-1}. NOTE: only quantification transitions.

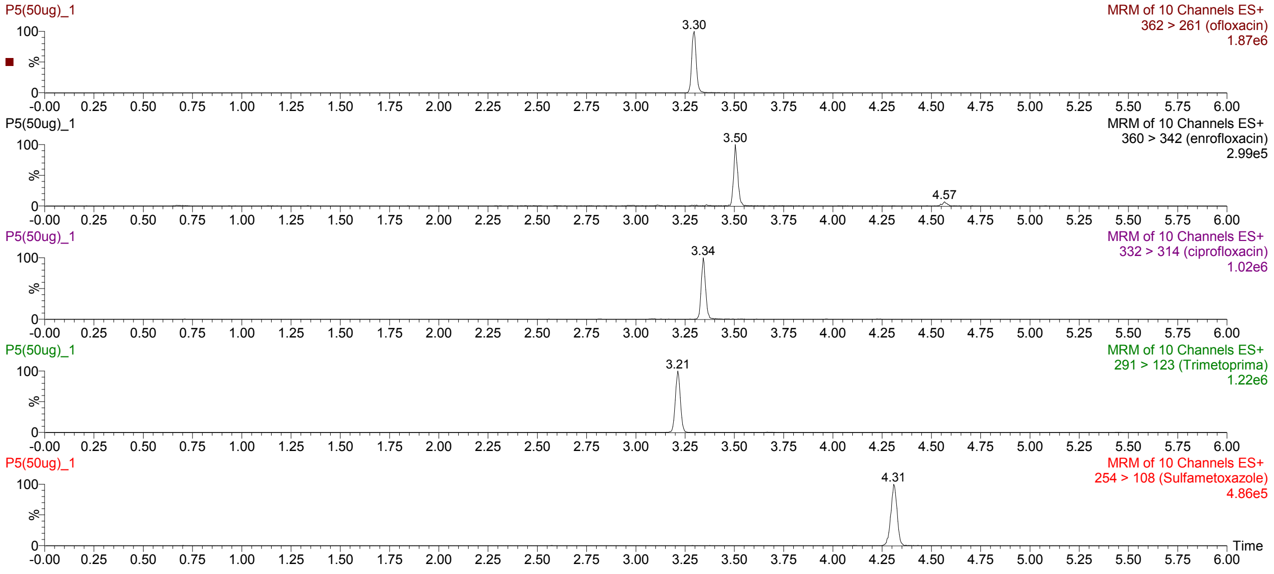

Supplement: Supplementary file 1 [file ao6c02775_si_001.pdf]
